# Supplementary material for: On–off conduction photoswitching in modelled spiropyran-based metal-organic frameworks
Source: Commun Chem. 2023 Dec 18;6:275. doi: 10.1038/s42004-023-01072-4 (PMC10728195; doi:10.1038/s42004-023-01072-4)
Supplement: Supplementary file 2 — Supplementary Information [file 42004_2023_1072_MOESM2_ESM.docx]

**Supplementary Information**

**On-Off Conduction Photoswitching in modelled Spiropyran-based Metal-Organic Frameworks**

Mersad Mostaghimi^1,§^, Helmy Pacheco Hernandez^1,§^, Yunzhe Jiang^2^, Wolfgang Wenzel^1^, Lars Heinke^2,*^ and Mariana Kozlowska^1,*^

^1^ Institute of Nanotechnology (INT), Karlsruhe Institute of Technology (KIT), Kaiserstraße 12, 76131 Karlsruhe, Germany

^2^ Institute of Functional Interfaces (IFG), Karlsruhe Institute of Technology (KIT), Kaiserstraße 12, 76131 Karlsruhe, Germany

^§^ Contributed equally

^*^ Corresponding author

**Description of the computational approach**


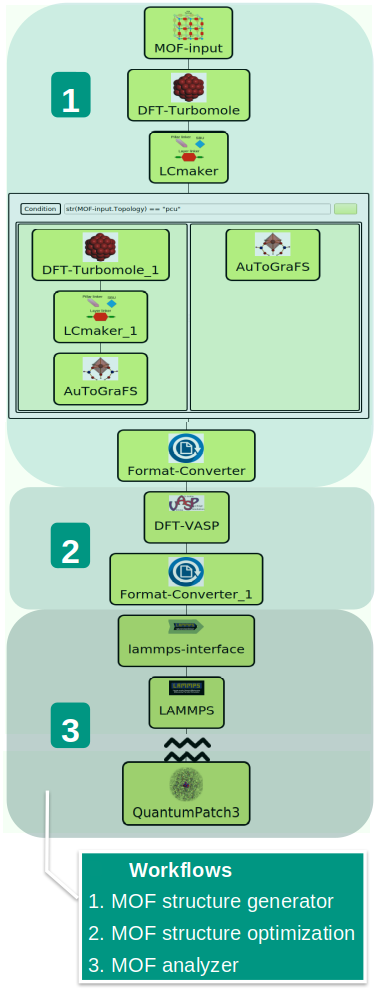


**Figure S1.** Workflow within the SimStack client to build (1), optimize (2), and analyze (3) MOF materials for automated structure-property predictions and design. Each module of the workflow, called Workflow active Node (WaNo) performs a particular type of calculation (see details if Ref.^1^) and data generated are automatically passed to the next step.

**Supplementary methodology description:**

Most of the calculations were performed using the workflow^1^ depicted in Figure S1. It can be divided in three essential parts:

**1) MOF structure generator**

In the *MOF-input* WaNo, the layer and pillar linkers are uploaded in the xyz format, and the desired metal (i.e. metal node type/topology) is selected. Files with predefined and optimized metal topology, available in the AuToGraFS library, are used^2^. For this study, we have use paddle-wheel Cu oxo-node called “Cu_pw6”. Either dabco or bipyridine were used as pillar linkers, coordinated to Cu oxo-node through the nitrogen atom (Cu⋯N); and either spiropyran or merocyanine (with different R-substitution) were used as layer linkers, i.e. coordinated to Cu through the oxygen atom from the carboxyl group (Cu⋯O). Next, the pillar and layer linkers are separately optimized, using Turbomole version 7.4.1, with the B3LYP functional and def2-TZVP basis set. Furthermore, the Grimme D3 dispersion correction is incorporated into the calculations. DFT optimization of these two linker types is running in *DFT-Turbomole* and *DFT-Turbomole_1* WaNo. This means that both linkers are calculated separately and are later connected to separate *LCmaker* WaNos, where linkers are prepared for the building of the MOF model using *AuToGraFS*. Since coordination to the metal node is different in both cases, different preparation algorithms were implemented. A more detailed explanation is described in Mostaghimi et al paper^1^. In general, LCmaker incorporates 'dummy atoms' within the linkers, which later guide AuToGraFS in joining the linkers with the metal node (Cu⋯N and Cu⋯O).

**2) MOF structure optimization:**

After the generation of the MOF structure, a geometry optimization of the periodic MOF structure based on plane wave DFT is performed using the VASP software version 5.4.4. In this study, DFT calculations were carried out within the *DFT-VASP* WaNo. The Perdew-Burke-Ernzerhof (PBE) functional was selected for the optimization process. A plane wave energy cutoff of 500 eV and a k-point grid with dimensions of 2x2x2 were used. The electron-ion interactions were described by the PAW (projector augmented wave) potentials. Full geometry optimization of Cu_2_(SP)_2_(dabco) and Cu_2_(MC)_2_(dabco) was performed using the conjugate gradient algorithm until the electronic self-consistent-loop reached convergence of 10^-4^ eV and the ionic relaxation loop of 0.01 eV. The Tkatchenko-Scheffler method with iterative Hirshfeld partitioning was used to include dispersion correction. The pre-optimization of all other MOFs, utilized as an input for MD simulations, was performed with 100 ionic steps with the break ionic relaxation criterion of -0.02 eV. Grimme D2 dispersion correction was used to accelerate pre-optimization.

After this step, two directions were taken over:

i) Dimers of the layer linkers were extracted from the optimized MOF and the calculation of the electronic coupling between HOMO and LUMO orbitals of extracted linkers as a function of separation distance were performed. This was done for DFT optimized Cu_2_(SP)_2_(dabco) and Cu_2_(MC)_2_(dabco) pillared MOFs.

ii) Results of *DFT-VASP* Wano were automatically used for performing MD simulations in Large-scale Atomic Molecular Massively Parallel Simulator (LAMMPS). For that, *Format-Converter_1* Wano was used to convert output files from VASP to input files of LAMMPS. Setup of MD simulations and preparation of necessary input files was done by *lammps-interface*, as implemented by Boyd et al^3^. A full explanation is given in part 3 of the workflow.

**3) MOF Analyzer**

In this section, the workflow can be customized based on the specific properties of the MOF under study. In our case, MD simulations of MOFs at 298 K were performed using the UFF4MOF force field^4^ using LAMMPS. For the equilibration part we used the Langevin thermostat^5^ and microcanonical ensemble (NVE) for 30 ps. It was followed by 10 ns MD production run in a canonical ensemble (NVT) and Nosé-Hoover thermostat^6^. The periodically repeated 4×4×4 MOF supercells were simulated with a timestep of 1 fs. All other parameters were taken as specified in lammps-interface, i.e. real unit with LJ interactions cut-off 12.5 Å^7^.

Such described workflow is available under <https://github.com/KIT-Workflows/PCU-MOF> . It can be downloaded and used via SimStack client using user-defined pillar- and layer-like linker. Note: software necessary to perform all calculations has to be pre-installed separately. WaNos, workflow, and SimStack clients have been made to manage various computational tasks, but they do not provide installations of software mentioned before.

MD trajectories were saved every 1000 steps, which resulted in 10300 frames. 1030 frames were analyzed using VMD software^8^. Equilibration data (first 30 frames) were not taken for further analysis. The detailed analysis of MD trajectories was necessary to enumerate the pair of layer linkers, representing a dimer used for further calculations (depicted in Figure 4). Since the enumeration of residues (defined via force field) is random, the pair of monomers had to be searched for all individual MOFs studied. 250 snapshots (every 20 ps) of the selected dimer (and trimer, in the case of Cu_2_(MC)_2_(dabco)) from the last 5 ns MD simulations were extracted using in-house script. They were later automatically hydrogenated (COO^-^ groups that were linked to Cu metal nodes in a MOF) using Atomic Simulation Environment (ASE)^9^ and openbabel^10^. For each of the snapshots, the electronic coupling between the molecules was calculated using equations 2-5 in the main body using Quantum Patch^11^. B3LYP functional with def2-SVP basis set and 7 equilibration steps for the self-consistent equilibration of the charge densities of all molecules were used for the calculation. This step was performed outside of the workflow depicted in Figure S1, however, can be coupled to the workflow too. It was presently not performed due to the lack of automation of MD data analysis step with the automated extraction of dimers.

The flowchart of the workflow, including information on steps done, is depicted in Figure S2.


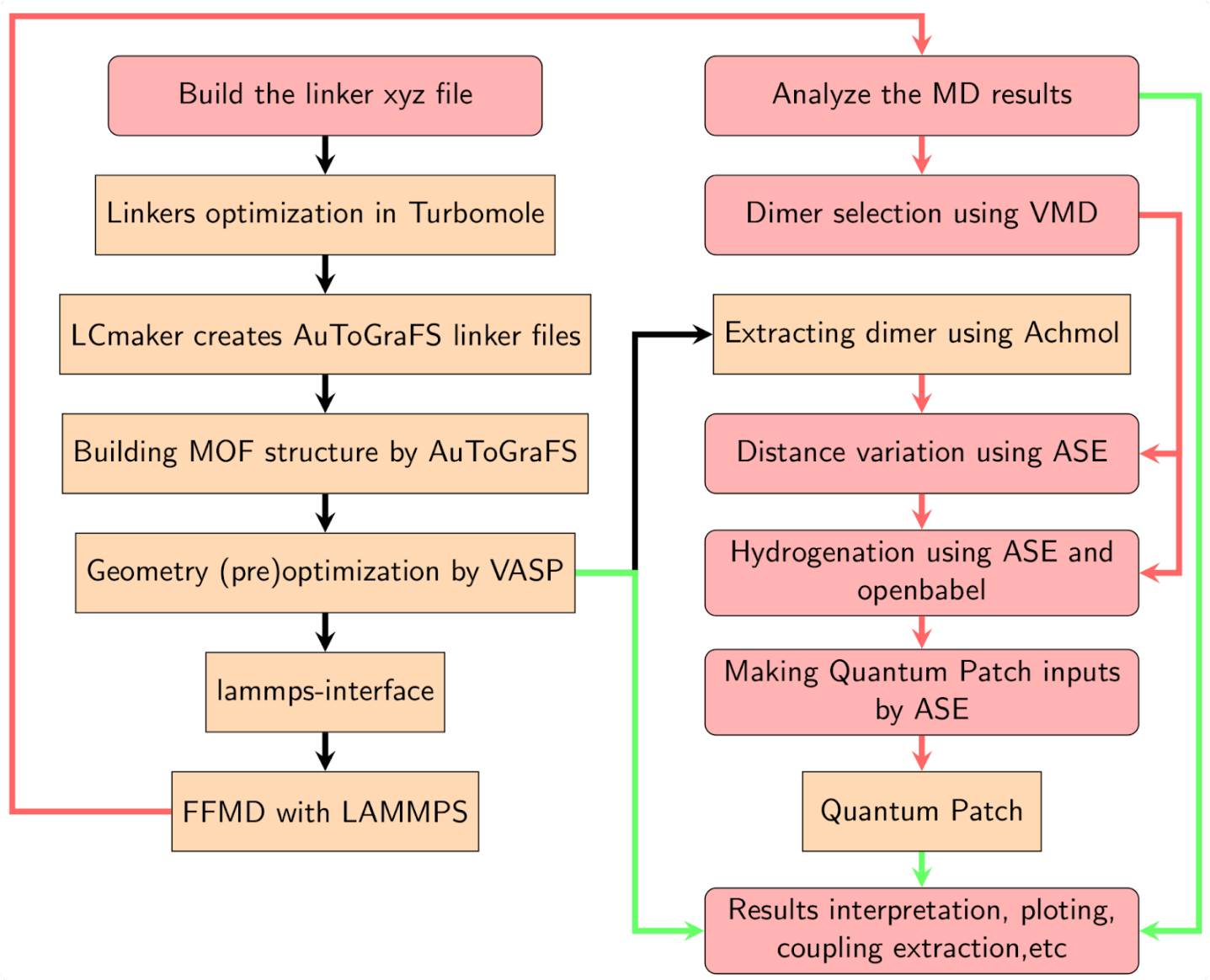


**Figure S2**. The flowchart of the calculations performed. Boxes in orange, followed by black arrows, are automated within the workflow in SimStack (see Figure S1), whereas boxes in red indicate steps that require manual inputs. The red arrows indicate in-house scripts used to automate some manual procedures. They involve the use of additional software like the Atomic Simulation Environment (ASE), openbabel and Visual molecular dynamics (VMD). The green arrows are results indicators. Two post-processing pathways after the dimer extraction from MD data were used in the present study: 1) without distance variation and calculation of electronic couplings in MD snapshots and 2) with distance variation applied to several MD averaged snapshots, followed by hydrogenation and QP input preparation step to calculate couplings.


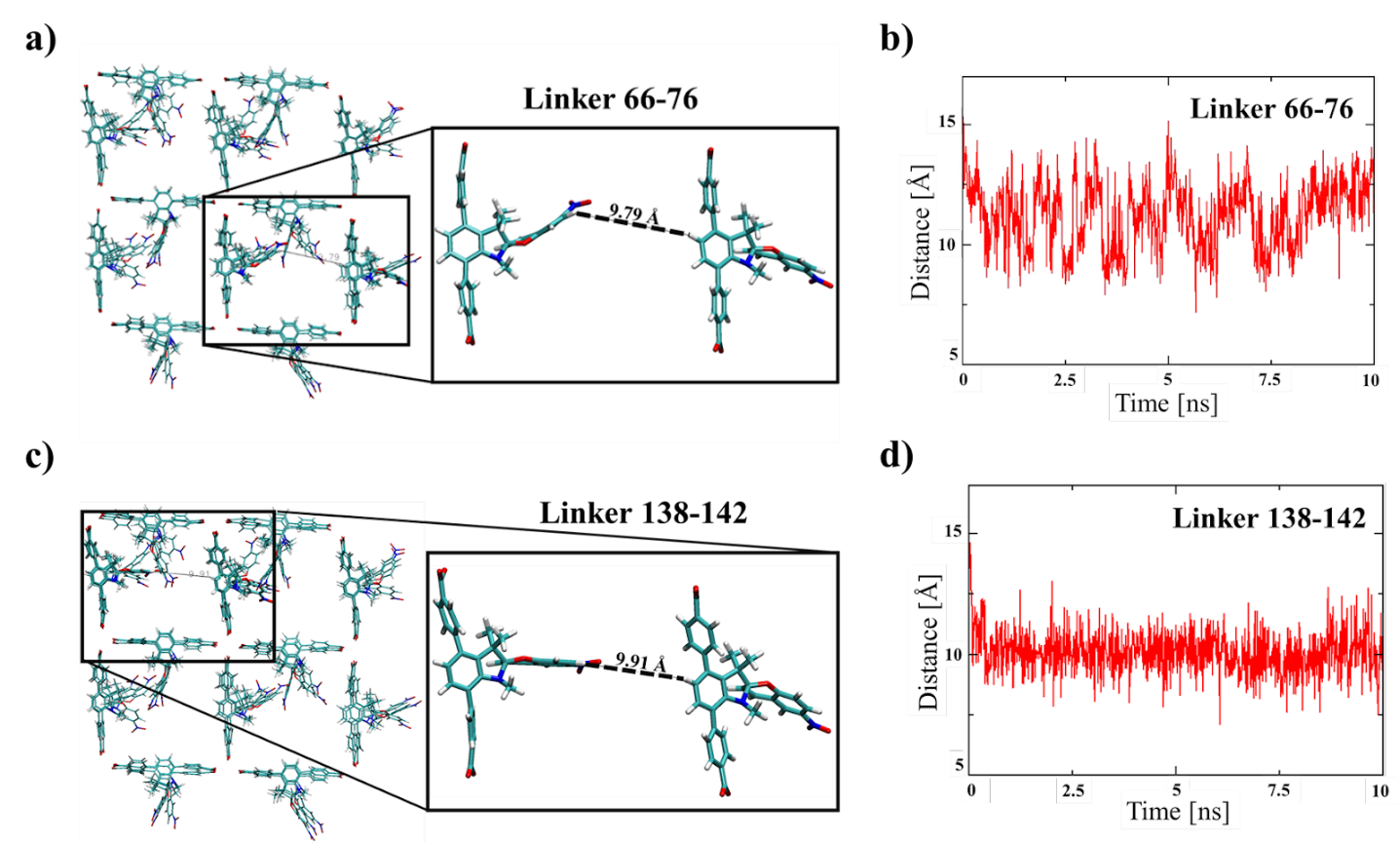


**Figure S3.** The visualization of the intermolecular distance between spiropyran-based layer linkers in the Cu_2_(SP)_2_(dabco) MOF: a) and c) distance between the closest atoms between two different linker pairs (MD snapshot at 5.7 ns). b) and d) time evolution of the intermolecular distance in MD at 298 K.


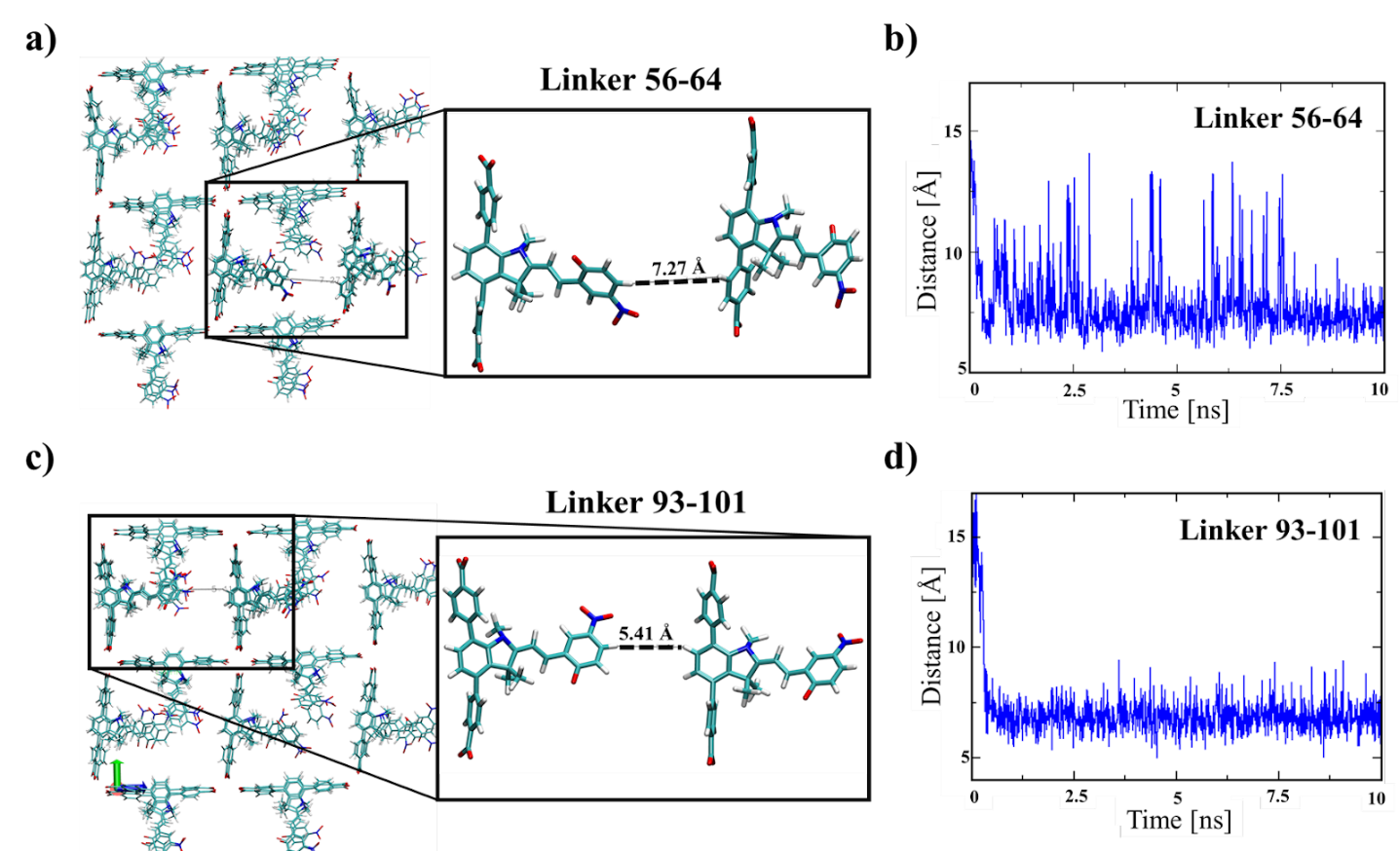


**Figure S4**. The visualization of the intermolecular distance between merocyanine-based layer linkers in the Cu_2_(MC)_2_(dabco) MOF: a) and c) distance between the closest atoms between two different linker pairs (MD snapshot at 5.7 ns). b) and d) time evolution of the intermolecular distance in MD at 298 K.


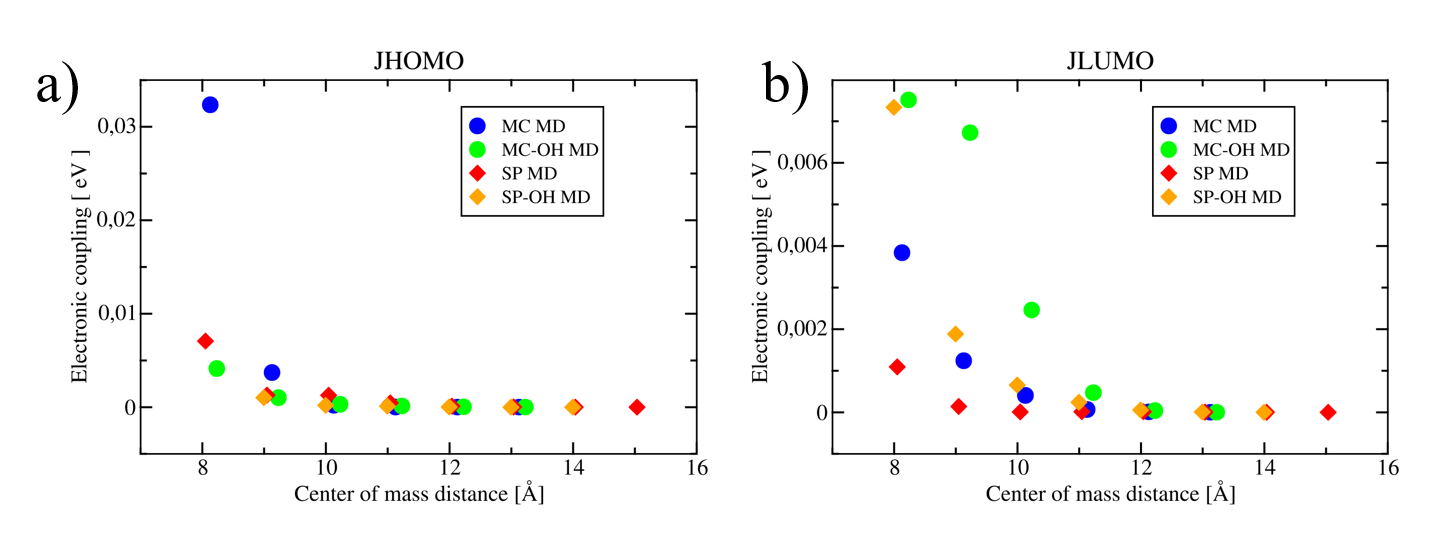


**Figure S5.** Dependence of the electronic coupling between a) HOMO and b) LUMO orbitals on the distance between parallel layer linkers in the pillared MOF (see scheme in Figure 3a,b). Distance screening from MD equilibrated structures of Cu_2_(**SP**)_2_(dabco), Cu_2_(**SP-OH**)_2_(dabco), Cu_2_(**MC**)_2_(dabco) and Cu_2_(**MC-OH**)_2_(dabco) are presented.


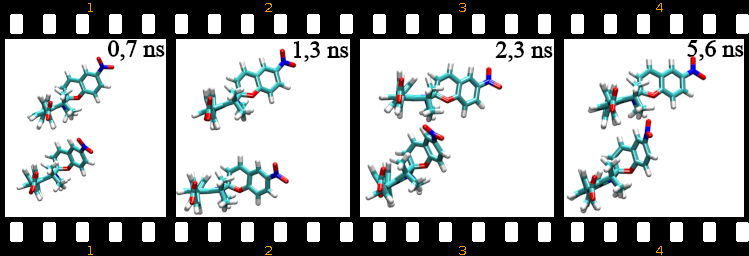


**Figure S6.** Selected snapshots of layer linkers extracted from the neighboring **Cu_2_(SP)_2_(dabco)** MOF layers. The corresponding timestamp from molecular dynamics simulations at 298K is given for clarity. Pillar linkers were omitted for visualization. Carbon, oxygen, nitrogen, hydrogen atoms are in cyan, red, blue and white, respectively.


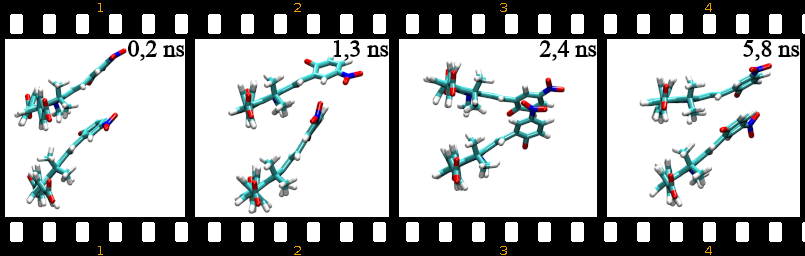


**Figure S7.** Selected snapshots of layer linkers extracted from the neighboring **Cu_2_(MC)_2_(dabco)** MOF layers. The corresponding timestamp from molecular dynamics simulations at 298K is given for clarity. Pillar linkers were omitted for visualization. Carbon, oxygen, nitrogen, hydrogen atoms are in cyan, red, blue and white, respectively.


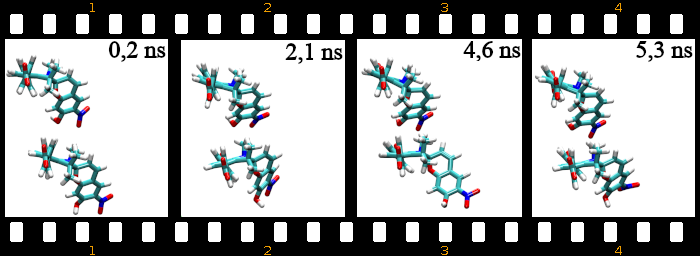


**Figure S8.** Selected snapshots of layer linkers extracted from the neighboring **Cu_2_(SP-OH)_2_(dabco)** MOF layers. The corresponding timestamp from molecular dynamics simulations at 298K is given for clarity. Pillar linkers were omitted for visualization. Carbon, oxygen, nitrogen, hydrogen atoms are in cyan, red, blue and white, respectively.


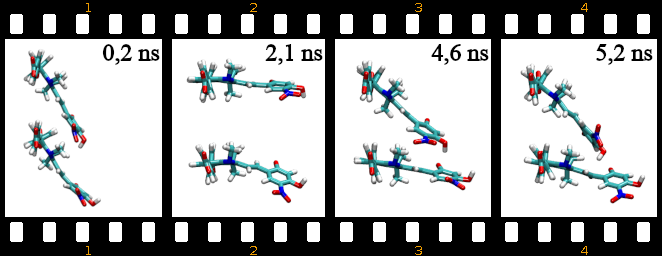


**Figure S9.** Selected snapshots of layer linkers extracted from the neighboring **Cu_2_(MC-OH)_2_(dabco)** MOF layers. The corresponding timestamp from molecular dynamics simulations at 298K is given for clarity. Pillar linkers were omitted for visualization. Carbon, oxygen, nitrogen, hydrogen atoms are in cyan, red, blue and white, respectively.


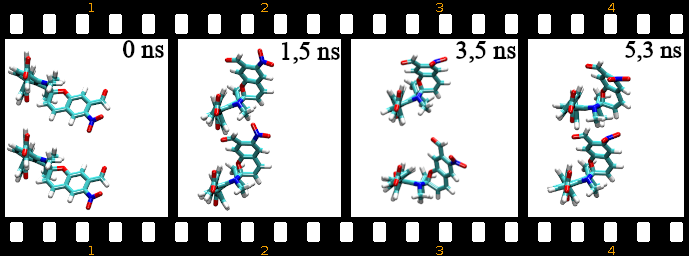


**Figure S10.** Selected snapshots of layer linkers extracted from the neighboring **Cu_2_(SP-CHO)_2_(dabco)** MOF layers. The corresponding timestamp from molecular dynamics simulations at 298K is given for clarity. Pillar linkers were omitted for visualization. Carbon, oxygen, nitrogen, hydrogen atoms are in cyan, red, blue and white, respectively.


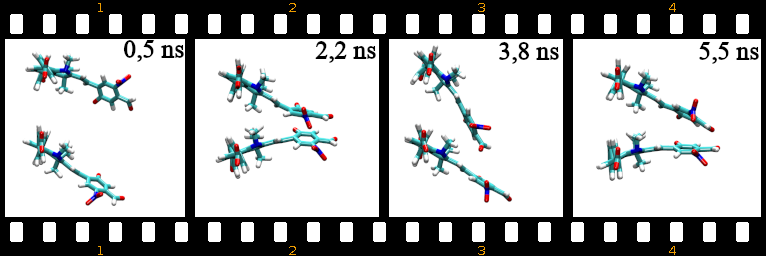


**Figure S11.** Selected snapshots of layer linkers extracted from the neighboring **Cu_2_(MC-CHO)_2_(dabco)** MOF layers. The corresponding timestamp from molecular dynamics simulations at 298K is given for clarity. Pillar linkers were omitted for visualization. Carbon, oxygen, nitrogen, hydrogen atoms are in cyan, red, blue and white, respectively.


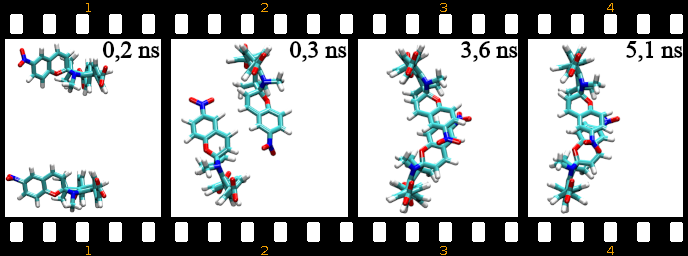


**Figure S12.** Selected snapshots of layer linkers extracted from the neighboring **Cu_2_(SP)_2_(bipy)** MOF layers. The corresponding timestamp from molecular dynamics simulations at 298K is given for clarity. Pillar linkers were omitted for visualization. Carbon, oxygen, nitrogen, hydrogen atoms are in cyan, red, blue and white, respectively.


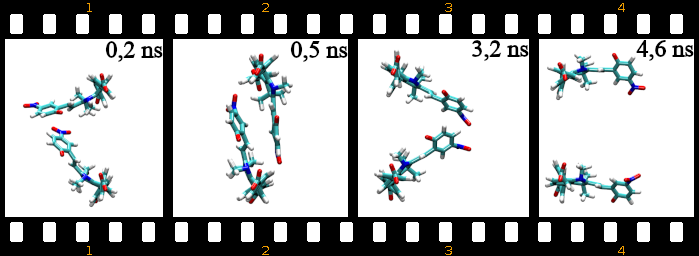


**Figure S13.** Selected snapshots of layer linkers extracted from the neighboring **Cu_2_(MC)_2_(bipy)** MOF layers. The corresponding timestamp from molecular dynamics simulations at 300K is given for clarity. Pillar linkers were omitted for visualization. Carbon, oxygen, nitrogen, hydrogen atoms are in cyan, red, blue and white, respectively.

**Table S1.** Approximate on-off conduction switching upon photoisomerization in spiropyran-based MOFs. The highest and average electronic coupling, *J*, between SP- and MC-based linkers in Cu_2_(SP)_2_(dabco) and Cu_2_(MC)_2_(dabco) MOFs in 250 snapshots from MD simulations (last 5 ns from a 10 ns MD production run). Electronic coupling is given in meV.

|  | **SP** | | **MC** | | **On-off ratio**  **p-conduction** | **On-off ratio**  **n-conduction** |
| --- | --- | --- | --- | --- | --- | --- |
|  | **HOMO** | **LUMO** | **HOMO** | **LUMO** |  |  |
| $\boldsymbol{J}_{\boldsymbol{highest}}$ | 19.9 | 0.24 | 159.4 | 55.7 | ~8^2^ | ~224^2^ |
| $\boldsymbol{J}_{\boldsymbol{average}}^{\boldsymbol{arithm}}$ | 1.15±2.03 | 0.36±0.39 | 26.79±27.77 | 23.25±20.26 | ~23^2^ | ~65^2^ |
| $\boldsymbol{J}_{\boldsymbol{average}}^{\boldsymbol{geom}}$ | 0.55*/4.02 | 0.16*/6.15 | 15.27*/3.75 | 14.34*/3.56 | - | - |


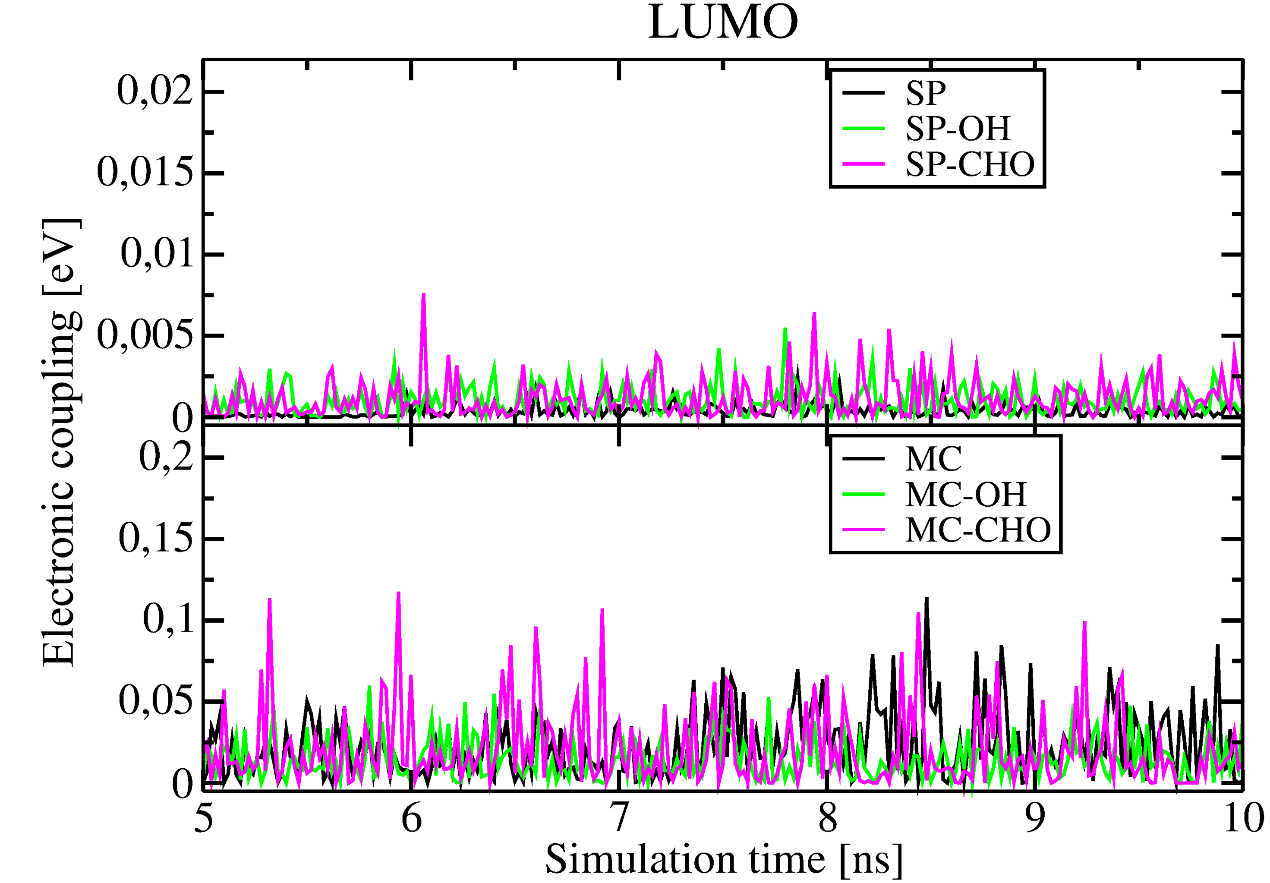


**Figure S14.** The time evolution of the electronic coupling between LUMO orbitals of unmodified SP and MC linkers in Cu2(SP)2(dabco) and Cu2(MC)2(dabco) (in black), and their OH- and CHO-modified analogs from Cu2(SP-OH)2(dabco) and Cu2(MC-OH)2(dabco) (in green), and Cu2(SP-CHO)2(dabco) and Cu2(MC-CHO)2(dabco) (in violet), respectively. Only direct electronic couplings are considered.


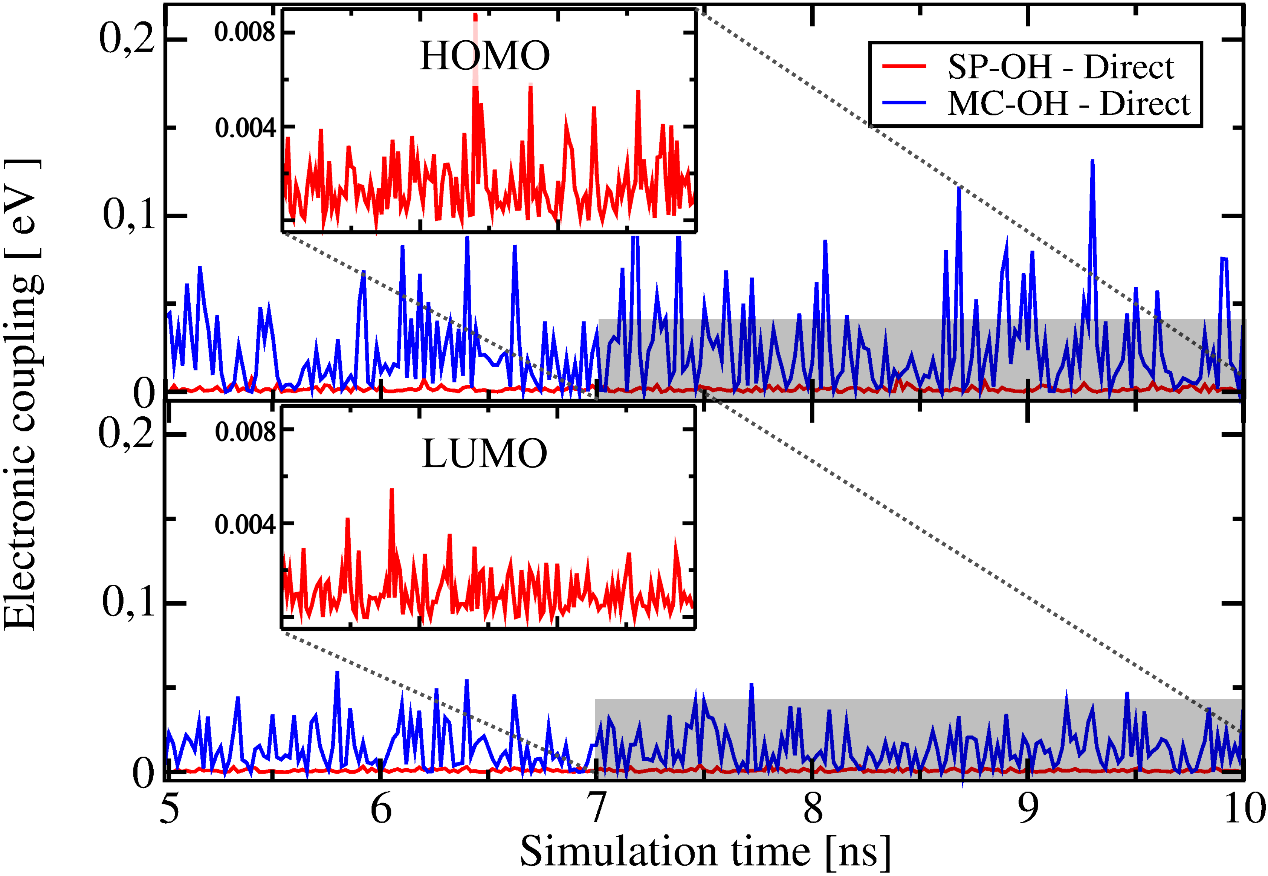


**Figure S15.** Electronic coupling between linkers in **Cu_2_(SP-OH)_2_(dabco) and** **Cu_2_(MC-OH)_2_(dabco)** MOFs.


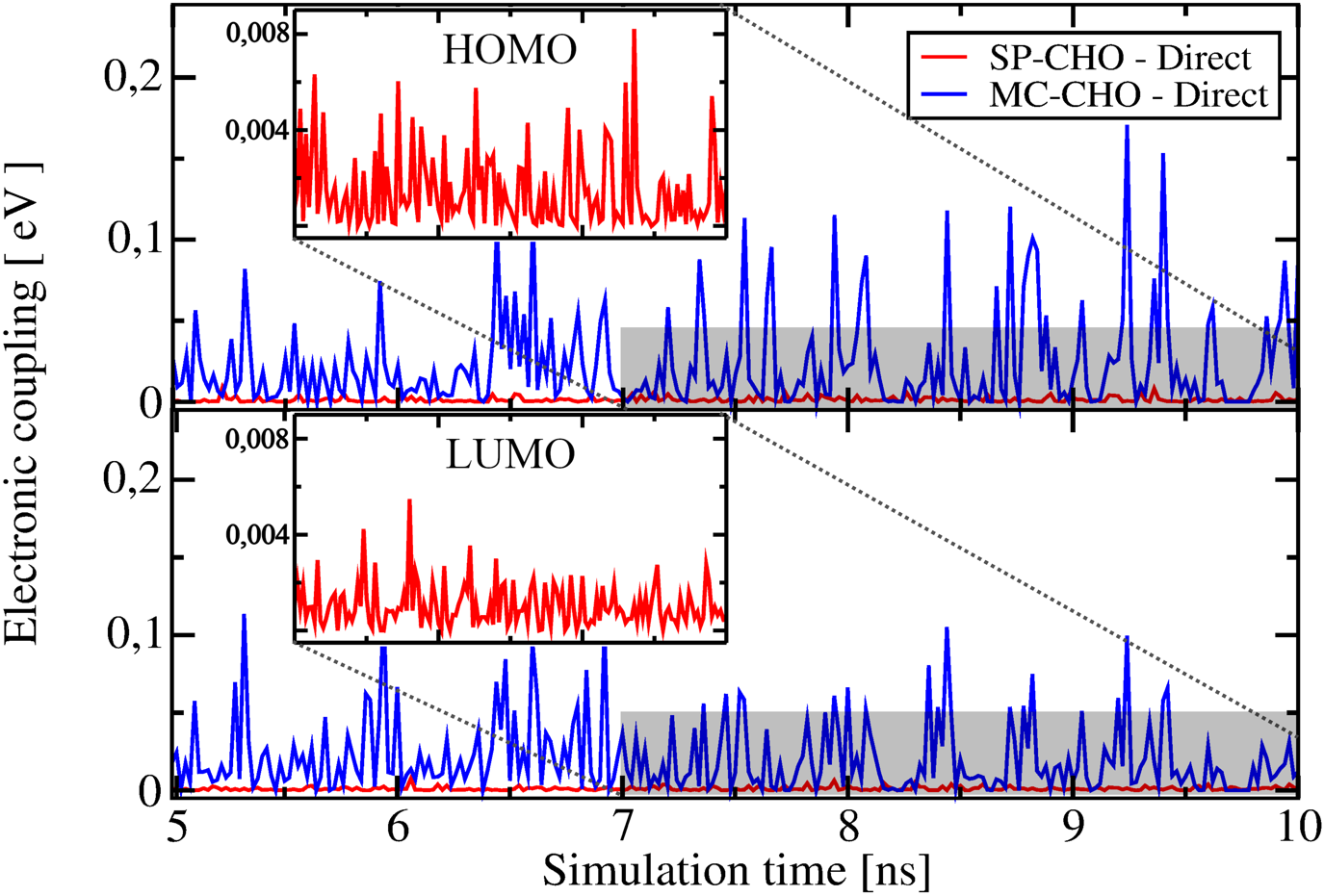


**Figure S16.** Electronic coupling between linkers in **Cu_2_(SP-CHO)_2_(dabco) and** **Cu_2_(MC-CHO)_2_(dabco)** MOFs.


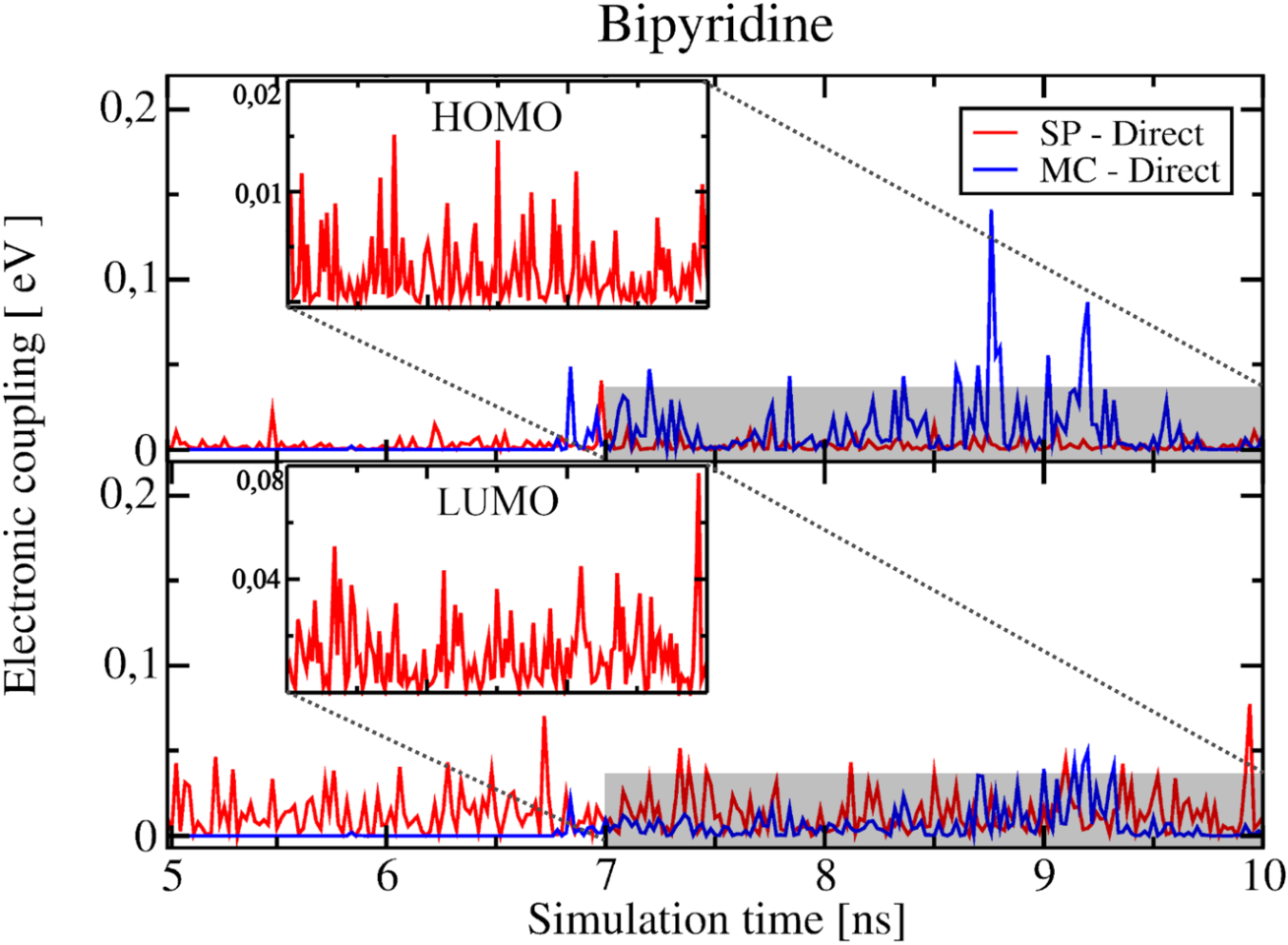


**Figure S17.** Electronic coupling between linkers in Cu_2_(SP)_2_(bipy) and Cu_2_(MC)_2_(bipy) MOFs with bipyridine as pillar.


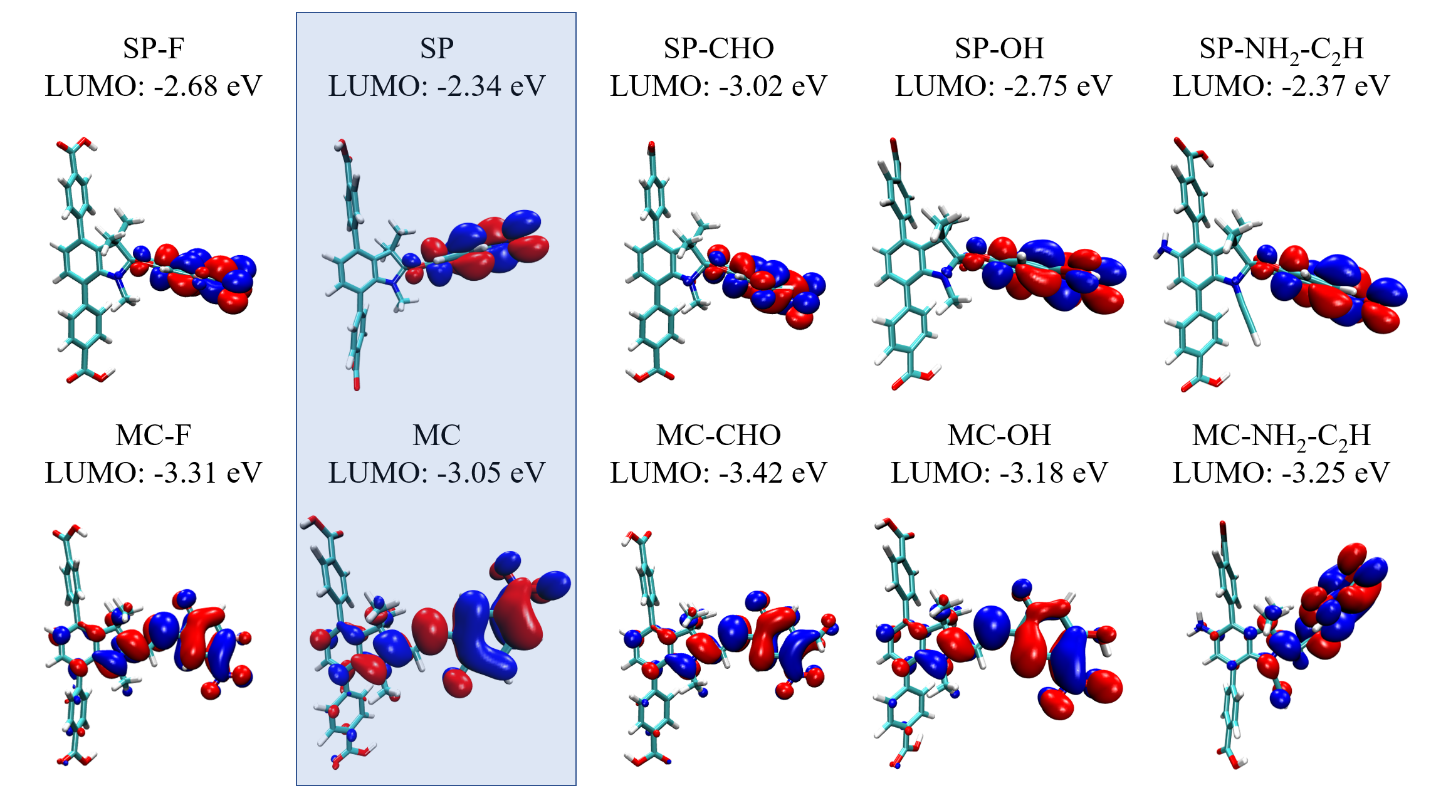


**Figure S18.** Visualization of LUMO orbitals of linkers considered in the present study (see Figure 2 for clarity). Orbitals are given with their respective orbital energy. Isovalue of 0.02 a.u. was used for visualization. Pillared MOFs made from unmodified SP and MC linkers are denoted with the blue color. MOFs with SP, MC, SP-OH, MC-OH, SP-CHO and MC-CHO layer linkers were simulated.

**Table S2.** Energy of frontier orbitals of spiropyran-based and merocyanine-based linkers, shown in Figure S17. Data of initial unmodified linkers, labeled as SP and MC over the text, are marked in grey. All values are in eV.

| **Functional groups** | **HOMO** | | **LUMO** | | **ΔHOMO_SP-MC_** | **ΔLUMO_SP-MC_** |
| --- | --- | --- | --- | --- | --- | --- |
|  | **SP** | **MC** | **SP** | **MC** |  |  |
| **R_1_= H**  R_2_= CH3  R_3_= H | -6.06 | -5.71 | -2.34 | -3.05 | -0.35 | +0.71 |
| **R_1_= OH**  R_2_= CH3  R_3_= H | -5.79 | -5.68 | -2.75 | -3.18 | -0.11 | +0.43 |
| **R_1_= CHO**  R_2_= CH3  R_3_= H | -6.03 | -6.01 | -3.02 | -3.42 | -0.02 | +0.40 |
| **R_1_= F**  R_2_= CH3  R_3_= H | -6.07 | -6.01 | -2.68 | -3.31 | -0.06 | +0.63 |
| R_1_= H  **R_2_= C_2_H**  **R_3_= NH_2_** | -5.35 | -5.66 | -2.37 | -3.25 | +0.31 | +0.88 |


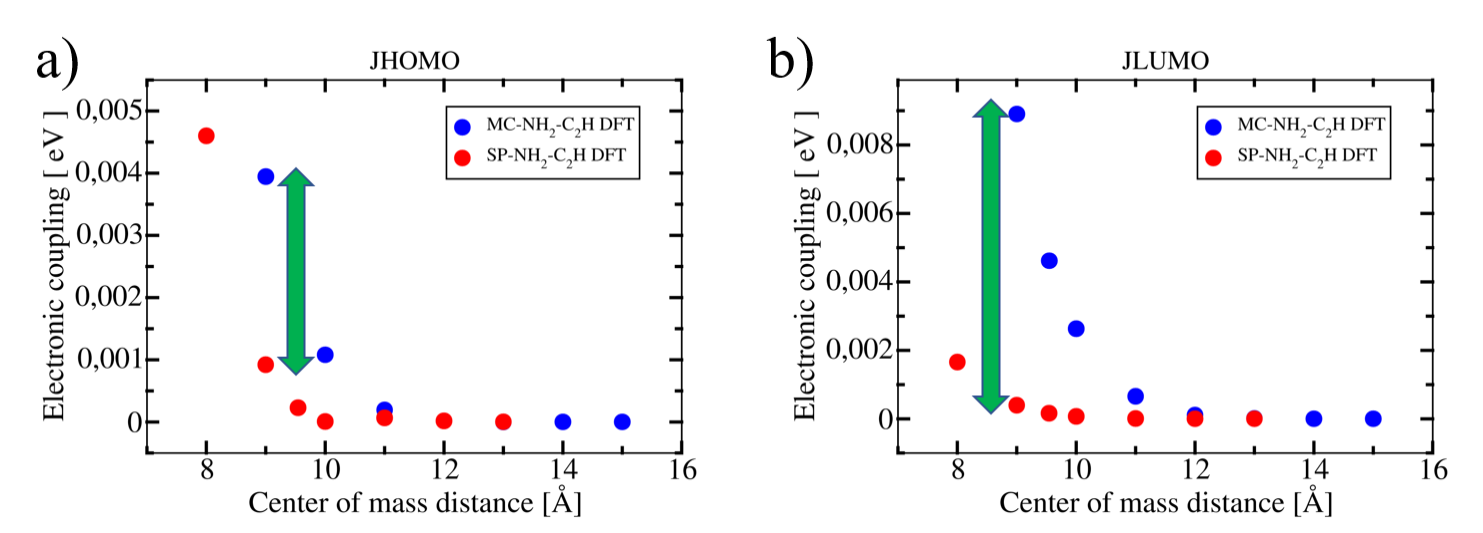


**Figure S19.** Dependence of the electronic coupling between a) HOMO and b) LUMO orbitals on the distance between parallel layer linkers in the pillared Cu_2_(SP-NH_2_-C_2_H)_2_(dabco) and Cu_2_(MC-NH_2_-C_2_H)_2_(dabco) MOF (see scheme in Figure 3a,b). Distance screening from DFT optimized structures is presented.

**Supplementary References**

1. Mostaghimi, M. *et al.* Automated Virtual Design of Organic Semiconductors Based on Metal-Organic Frameworks. *Front. Mater.* **9**, 840644 (2022).

2. Addicoat, M. A., Coupry, D. E. & Heine, T. AuToGraFS: Automatic Topological Generator for Framework Structures. *J. Phys. Chem. A* **118**, 9607–9614 (2014).

3. Boyd, P. G., Moosavi, S. M., Witman, M. & Smit, B. Force-Field Prediction of Materials Properties in Metal-Organic Frameworks. *J. Phys. Chem. Lett.* **8**, 357–363 (2017).

4. Addicoat, M. A., Vankova, N., Akter, I. F. & Heine, T. Extension of the Universal Force Field to Metal–Organic Frameworks. *J. Chem. Theory Comput.* **10**, 880–891 (2014).

5. Schneider, T. & Stoll, E. Molecular-dynamics study of a three-dimensional one-component model for distortive phase transitions. *Phys. Rev. B* **17**, 1302–1322 (1978).

6. Hoover, W. G. Canonical dynamics: Equilibrium phase-space distributions. *Phys. Rev. A* **31**, 1695–1697 (1985).

7. Allen, M. P. & Tildesley, D. J. *Computer Simulation of Liquids*. (Oxford University Press, 2017).

8. Humphrey, W., Dalke, A. & Schulten, K. VMD: Visual molecular dynamics. *J. Mol. Graph.* **14**, 33–38 (1996).

9. Larsen, A. H. *et al.* The atomic simulation environment—a Python library for working with atoms. *J. Phys. Condens. Matter* **29**, 273002 (2017).

10. O’Boyle, N. M. *et al.* Open Babel: An open chemical toolbox. *J. Cheminformatics* **3**, 33 (2011).

11. Friederich, P., Symalla, F., Meded, V., Neumann, T. & Wenzel, W. Ab initio treatment of disorder effects in amorphous organic materials: toward parameter free materials simulation. *J. Chem. Theory Comput.* **10**, 3720−3725 (2014).
